# Efficacy and safety of indocyanine green-fluorescence imaging guided liver resection: a single-arm prospective cohort study

**DOI:** 10.1007/s00423-024-03602-7

**Published:** 2025-01-11

**Authors:** Hidetoshi Gon, Satoshi Omiya, Shohei Komatsu, Nobuaki Yamasaki, Sae Murakami, Kenji Fukushima, Takeshi Urade, Daisuke Tsugawa, Hiroaki Yanagimoto, Hirochika Toyama, Masahiro Kido, Takumi Fukumoto

**Affiliations:** 1 Department of Surgery, Division of Hepato-Biliary-Pancreatic Surgery, Kobe University Graduate School of Medicine, Kobe, Hyogo Japan; 2 Clinical and Translational Research Center, Kobe University Hospital, Kobe, Hyogo Japan

**Keywords:** Indocyanine green-fluorescence imaging, Liver tumor, Liver resection, Surgical outcome

## Abstract

**Purpose:**

This study aimed to evaluate the efficacy of indocyanine green (ICG)-fluorescence imaging for the identification of hepatic boundaries during liver resection and its advantages in surgical outcomes over conventional methods.

**Methods:**

This prospective, exploratory, single-arm clinical trial included 47 patients with liver tumors who underwent liver resection using ICG-fluorescence imaging (ICG-LR) between 2019 and 2020. The primary outcome measure was the successful identification of hepatic boundaries during liver resection, from the perspective of both the hepatic surface and intrahepatic boundary, using ICG-fluorescence imaging. The secondary outcomes comprised surgical outcomes. Using propensity score matching (PSM), the surgical outcomes were subsequently compared between the ICG-LR group and patients who underwent conventional liver resection (C-LR, *n* = 100) between 2017 and 2018.

**Results:**

Hepatic boundaries were successfully identified in 28 patients (60%; 95% confidence interval, 45–72%), including 21 and 7 who underwent anatomical and non-anatomical liver resection, respectively. After PSM, 40 patients were included in each of the ICG-LR and C-LR groups. The surgical outcomes were similar between the groups. Subsequently, surgical outcomes were compared between the groups focusing on anatomical liver resection. After PSM, 21 patients were included in each group. The ICG-LR group had a lower rate of Clavien–Dindo grade ≥ IIIa complications (0% vs. 24%; *P* = 0.017), including ascites and bile leak, and a shorter hospital stay (12 vs. 14 days, *P* = 0.041) than the C-LR group did.

**Conclusion:**

ICG-fluorescence imaging could be used to recognize hepatic boundaries during liver transection. Additionally, ICG-LR may be useful in preventing severe liver-associated complications.

**Trial registration number:**

This study is registered at the UMIN Clinical Trials Registry: UMIN0000180139 and Japan Registry of Clinical Trials: jRCT1051180070. The Registration Data Set is available at https://jrct.niph.go.jp/.

**Supplementary Information:**

The online version contains supplementary material available at 10.1007/s00423-024-03602-7.

## Introduction

Liver resection remains the mainstay of treatment for liver tumors and is commonly performed in patients with preserved liver function [1–3]. It has been conventionally performed—guided by anatomical structures, including the Glissonean branches or pedicles and hepatic vein—using intraoperative ultrasonography. However, owing to the three-dimensional shape of the hepatic structure, the two-dimensional image that ultrasonography provides may not always be sufficient as a guiding tool for liver resection, especially for less experienced surgeons. Indocyanine green (ICG)-fluorescence imaging is currently available as an additional method for navigation during liver resection, and its usefulness is recognized worldwide [4–9]. This technique allows the emphasized recognition of liver tumors and hepatic boundaries during liver resection [10]. Of note, ICG-fluorescence imaging has the potential to identify intrahepatic boundaries during parenchymal transection, demonstrating an appropriate liver transection line in a three-dimensional space [11, 12]. Nevertheless, the advantages of liver resection guided by superficial and intrahepatic boundaries visualized using ICG-fluorescence imaging compared to conventional liver resection (C-LR) have not been fully investigated. Precise parenchymal resection may minimize the ischemic area of the remnant liver tissue, possibly leading to favorable postoperative outcomes, including less ischemic change in the liver and associated complications. Therefore, through comprehensive evaluation of the ICG-fluorescence imaging technique, the surgical outcomes of both liver resection using ICG-fluorescence imaging (ICG-LR) and C-LR should be compared to demonstrate the real advantages of using ICG-fluorescence imaging during liver resection.

To this end, this prospective study evaluated the efficacy of ICG-LR in identifying superficial and intrahepatic boundaries during liver resection, and further investigated the actual advantages in terms of surgical outcomes of ICG-LR over C-LR in patients with liver tumors.

## Materials and methods

### Trial design

We conducted a prospective, single-arm, exploratory clinical trial to investigate the efficacy and safety of ICG-LR.

### Patients

Patients were recruited from the Kobe University Hospital. The study protocol was approved by the Kobe University Clinical Research Ethical Committee (approval number: CRB5180009). The trial was registered in the Japan Registry of Clinical Trials (jRCT) (registration number: jRCT1051180070). This study was conducted according to the ethical standards of the 1964 Declaration of Helsinki and its subsequent amendments.

Patients who were scheduled to undergo resection of liver tumors at Kobe University between 2019 and 2020 were recruited. The inclusion criteria were as follows: male or female patients with liver tumors, aged ≥ 20 years, scheduled for elective liver resection, with preserved liver function and the ability to understand the nature of the study procedures, and willing to participate and provided voluntary written consent. The exclusion criteria were liver insufficiency, known ICG hypersensitivity, pregnancy or breastfeeding, and the inability to understand the nature of the study procedure.

### Intervention

This study was conducted in accordance with previously reported procedures [13]. Briefly, ICG was injected intravenously at a dose of 0.5 mg/kg body weight within 2 days preoperatively. Intraoperatively, we initially observed the hepatic surface using ICG-fluorescence imaging to detect liver tumors. For anatomical liver resection, after identifying and clamping the portal pedicle corresponding to the hepatic area to be removed, additional ICG was injected intravenously at a dose of 0.5 mg/kg body weight to identify the boundaries of the hepatic area (negative staining technique) [14, 15]. During parenchymal resection, the demarcation between fluorescing and non-fluorescing areas was assumed to be the boundaries of the hepatic regions. Demarcation was checked at appropriate intervals during parenchymal resection. For non-anatomical liver resection, we used fusion ICG-fluorescence images after dissecting the corresponding Glissonean branches for the hepatic area to be removed according to the concept of cone unit resection [16]. Parenchymal resection was performed using the clamp-crushing technique. The Pringle maneuver was performed to control blood loss during parenchymal resection. During the study period, all surgeries were performed or supervised by a single, highly skilled laparoscopic surgeon to minimize technical bias.

### Outcome measures

The primary endpoint was the successful identification of the hepatic boundaries using ICG-fluorescence imaging. The assessment methodology has been previously described [13]. Briefly, we assumed that identification was successful when the boundaries were identified on both the hepatic and transection surfaces. The hepatic boundaries at the surface were deemed successfully identified by ICG-fluorescence imaging when the demarcation between fluorescing and non-fluorescing areas was consistent with the ischemic demarcation area observed by clamping the Glissonean pedicles or branches feeding on the tumor site. For the transection surface, the demarcation between the fluorescing and non-fluorescing areas was assumed to be the hepatic boundaries (Supplementary Fig. 1). We divided the time taken to perform parenchymal resection into three equal intervals and evaluated the identification of hepatic boundaries at each interval. The identification of hepatic boundaries was considered successful when the following conditions were met: (1) the demarcation between fluorescing and non-fluorescing areas was identified in > 80% of the transected area and (2) the condition of (1) was observed in two or more of the three intervals.

The secondary endpoints were the successful identification of liver tumors by ICG-fluorescence imaging, postoperative liver functional indicators, surgical outcomes, and 1-year recurrence-free survival (RFS). Postoperative complications were graded as previously described [13]. In this study, the following postoperative complications were classified as liver-associated: ascites, biliary leakage, and intra-abdominal abscess. The 1-year RFS was analyzed only in patients with hepatocellular carcinoma (HCC), while that of patients with other tumor types was not analyzed owing to the small sample size. RFS time was defined as the time from the date of surgery until the first recurrence thereafter. Subsequently, we compared the surgical outcomes between the ICG-LR and C-LR groups to investigate the impact of successful identification of hepatic boundaries during liver resection on surgical outcomes. In this analysis, successful and unsuccessful cases of hepatic boundary identification were included in the ICG-LR group. The C-LR group included patients who had undergone C-LR between 2017 and 2018 at our institution as historical controls—during this period, the ICG-fluorescence imaging technique had not yet been used. Surgical outcomes were also compared between the groups by focusing on the cases in which anatomical liver resection were performed. The safety endpoint was the frequency of adverse events.

### Sample size calculation

The original target sample size was 110 [13]. However, analyses were performed only in 47 patients who were included in this study by the registration deadline.

### Data collection

The methods of data collection have been previously described [13].

### Statistical analysis

Analysis was performed after data lock, following the administration of the study drug to all participants. All statistical analyses were performed using the JMP software, version 17.0.0 (SAS Institute, Inc., Cary, NC, USA).

Continuous variables are expressed as medians and interquartile ranges unless indicated otherwise, while categorical variables are expressed as absolute numbers (percentages). Differences between groups were evaluated using the Mann–Whitney U or chi-square tests. The proportions and 95% confidence intervals (CIs) for both the rate of identification of hepatic boundaries and liver tumors were estimated using a binomial distribution.

When we compared the surgical outcomes between the ICG-LR and C-LR groups, a 1:1 propensity score matching (PSM) analysis was performed to mitigate potential confounders and selection bias between the groups. The following factors were included in the regression model: age, sex, underlying liver disease, preoperative serum aspartate aminotransferase [AST] and alanine aminotransaminase [ALT] levels, serum albumin level, serum total bilirubin level, prothrombin time, platelet count, tumor size, tumor number, macrovascular invasion, and type of liver resection. The same procedure of 1:1 PSM was used for comparisons between the groups when focusing on anatomical liver resection.

Subgroup analyses were performed on patients with HCC to explore the differences in prognostic outcomes between the ICG-LR and C-LR groups. RFS was estimated using the Kaplan–Meier method and compared between the ICG-LR and C-LR groups using the log-rank test in the entire and matched cohorts. For this matched analysis, in addition to the abovementioned factors, serum alpha-fetoprotein levels were included in the regression model. Statistical significance was set at *P* < 0.05.

## Results

### Patient background characteristics and surgical factors

The background characteristics of the 47 patients included in this study are summarized in Table 1. The median patient age was 73 years. There were 35 (74%) and 12 (26%) male and female patients, respectively. Liver function assessment indicated that all 47 patients had a Child–Pugh classification of grade A. In terms of tumor factors, 34 patients (72%) were diagnosed with HCC, whereas 13 patients (28%) were diagnosed with other types of tumors, including metastasis and intrahepatic cholangiocarcinoma. The median tumor size was 3.5 cm. Eleven of the 47 patients (23%) had multiple liver tumor lesions.

**Table 1:** Patient background characteristics included in this study

		*Total*, n = *47*
General background		
Age (y) §		73 (69–80)
Sex, n	Male / Female	35 (74) / 12 (26)
BMI (kg/m^2^) §		22.6 (20.7–24.8)
Performance status, n	0 / 1 / 2 / 3	41 (87) / 5 (11) / 1 (2) / 0 (0)
Underlying liver disease, n	HB, HC / non-B non-C	26 (55) / 21 (45)
Child-Pugh classification, n	A / B	47 (100) / 0 (0)
Blood examination		
AST (IU/L) §		25 (22–47)
ALT (IU/L) §		25 (15–53)
Serum albumin (g/dL) §		4.1 (3.8–4.4)
Serum total bilirubin (mg/dL) §		0.7 (0.6–1.0)
Prothrombin time (%) §		102 (93–107)
Platelet count (× 10^3^mm^3^) §		20.4 (15.8–23.9)
ICG R15 (%) §		9.8 (7.1–13.4)
Tumor factor		
Diagnosis, n	HCC / Metastasis / ICC / others	34 (72) / 6 (13) / 3 (6) / 4 (9)
Tumor size (cm) §		3.5 (2.5–5.5)
Tumor number, n (%)	1 / ≥ 2	36 (77) / 11 (23)
Macrovascular invasion, n		7 (15)

Values in parentheses are percentages unless indicated otherwise; §values are median (interquartile range)

ALT, alanine transaminase; AST, aspartate aminotransferase; BMI, body mass index; HB, hepatitis B; HC, hepatitis C; HCC, hepatocellular carcinoma; ICC, intrahepatic cholangiocarcinoma; ICGR15, indocyanine green retention time at 15 min; non-B non-C, nonhepatitis B and nonhepatitis C

Surgical factors, including the success rate of hepatic boundary identification, are presented in Table 2. Twenty-nine patients (62%) underwent anatomical liver resection. Laparoscopic and open liver resection were performed in 38 (81%) and 9 (19%) patients, respectively. The median operative time and blood loss were 405 min and 120 mL, respectively. The hepatic boundaries were successfully identified in 28 patients (60%; 95% CI, 45–72%). Liver tumors were successfully identified in 28 patients (60%; 95% CI, 45–72%). Four patients (9%) had postoperative Clavien–Dindo grade ≥ IIIa complications. No patient experienced adverse events related to ICG injections.

**Table 2:** Patient surgical factors included in this study

		*Total*, n = *47*
Type of liver resection, n	Non-anatomical	18 (38)
	Anatomical	29 (62)
	Segmentectomy	12
	Sectionectomy	10
	Major resection	7
Surgical procedure, n	Open / laparoscopic	9 (19) / 38 (81)
Surgical outcomes		
Operation time (min) §		405 (280–504)
Blood loss (ml) §		120 (0–260)
Blood transfusion, n		3 (6)
Weight of resected liver (g) §		140 (65–224)
Surgical margin, n	Positive cases	3 (6)
ICG fluorescence technique, n		
Identification of tumors	Success	28 (60)
Identification of hepatic boundaries	Success	28 (60)
Postoperative blood examinations		
AST (IU/L) §		525 (292–693)
ALT (IU/L) §		429 (226–631)
Serum albumin (g/dL) §		2.6 (2.4–2.9)
Serum total bilirubin (mg/dL) §		1.4 (0.9–2.0)
Prothrombin time (%) §		72 (60–80)
Platelet count (10^3^/mm^3^) §		11.5 (9.3–16.2)
Postoperative outcomes		
Overall complication, n		17 (36)
Clavien-Dindo grade ≥ IIIa		4 (9)
Hospital stay (days) §		11 (9–14)

Values in parentheses are percentages unless indicated otherwise; §values are median (interquartile range)

ALT, alanine transaminase; AST, aspartate aminotransferase; ICG, indocyanine green

### Comparison of patient backgrounds and surgical factors between the ICG-LR and C-LR groups

Tables 3 and 4 present the comparisons of the baseline characteristics and surgical factors between the ICG-LR and C-LR groups, respectively. In the entire cohort, there was a significant difference in the diagnosis of liver tumors between the groups (Table 3; *P =* 0.006). Regarding surgical factors, the ICG-LR group had a significantly lower Clavien–Dindo grade ≥ IIIa complication rate than did the C-LR group (Tables 4 and 9% vs. 15%; *P* = 0.007). After PSM, 40 patients were included in each group. There were no significant differences in patient background characteristics between the ICG-LR and C-LR groups (Table 3). The surgical outcomes were similar between the groups (Table 4).

**Table 3:** Comparison of patient backgrounds between the ICG-LR and C-LR groups before and after PSM

		Before PSM				After PSM		
		ICG-LR n = 47	C-LR n = 100	P value		ICG-LR n = 40	C-LR n = 40	P value
General background								
Age (y) §		73 (69–80)	70 (65–76)	0.228		72 (68–79)	70 (66–75)	0.835
Sex, n	Male / Female	35 (74) / 12 (26)	77 (77) / 23 (23)	0.738		31 (78) / 9 (22)	33 (83) / 7 (17)	0.576
BMI (kg/m^2^) §		22.6 (20.7–24.8)	23.2 (20.7–26.1)	0.335		22.7 (20.8–25.0)	23.7 (20.6–25.7)	0.564
Performance status, n	0	41 (87)	90 (90)	0.869		35 (88)	36 (90)	0.925
	1	5 (11)	8 (8)			4 (10)	3 (8)	
	2	1 (2)	2 (2)			1 (2)	1 (2)	
Underlying liver disease, n	HB, HC	26 (55)	45 (45)	0.243		20 (50)	20 (50)	1.000
	non-B non-C	21 (45)	56 (56)			20 (50)	20 (50)	
Child-Pugh classification, n	A / B	28 (100) / 0 (0)	93 (93) / 7 (7)	0.063		40 (100) / 0 (0)	38 (95) / 2 (5)	0.152
Blood examination								
AST (IU/L) §		25 (22–47)	27 (21–36)	0.866		26 (21–47)	27 (21–37)	0.859
ALT (IU/L) §		25 (15–53)	24 (16–38)	0.778		26 (17–52)	25 (17–39)	0.821
Serum albumin (g/dL) §		4.1 (3.8–4.4)	4.0 (3.7–4.4)	0.389		4.2 (3.8–4.4)	4.2 (3.9–4.4)	0.632
Serum total bilirubin (mg/dL) §		0.7 (0.6–1.0)	0.6 (0.5–0.9)	0.182		0.7 (0.6–0.9)	0.8 (0.5–1.2)	0.843
Prothrombin time (%) §		102 (93–107)	99 (88–104)	0.063		102 (92–108)	101 (92–106)	0.513
Platelet count (10^3^/mm^3^) §		20.4 (15.8–23.9)	19.3 (15.2–27.0)	0.993		20.8 (15.8–24.2)	18.8 (13.9–24.4)	0.381
ICGR15 (%) §		9.8 (7.1–13.4)	10.4 (7.4–15.7)	0.392		9.2 (6.9–12.9)	9.2 (7.1–13.2)	0.683
Tumor factor								
Diagnosis, n	HCC	34 (72)	74 (74)	0.006		27 (67)	27 (67)	0.129
	Metastatic tumor	6 (13)	24 (24)			6 (15)	11 (28)	
	ICC	3 (6)	2 (2)			3 (8)	2 (5)	
	Others	4 (9)	0 (0)			4 (10)	0 (0)	
Tumor size (cm) §		3.5 (2.5–5.5)	3.8 (2.5–5.6)	0.652		4.3 (2.5–5.6)	3.3 (2.6–5.0)	0.497
Tumor number, n	1 / ≥ 2	36 (77) / 11 (23)	64 (64) / 36 (36)	0.121		29 (72) / 11 (28)	27 (67) / 13 (33)	0.625
Macrovascular invasion, n		7 (15)	15 (15)	0.987		7 (17)	7 (17)	1.000

Values in parentheses are percentages unless indicated otherwise; §values are median (interquartile range)

ALT, alanine transaminase; AST, aspartate aminotransferase; BMI, body mass index; C-LR, conventional liver resection; HB, hepatitis B; HC, hepatitis C; HCC, hepatocellular carcinoma; ICC, intrahepatic cholangiocarcinoma; ICGR15, indocyanine green retention rate at 15 min; ICG-LR, liver resection with ICG-fluorescence imaging; NA, not applicable; non-B non-C, nonhepatitis B and nonhepatitis C; PSM, propensity score matching

**Table 4:** Comparison of surgical factors between the ICG-LR and C-LR groups before and after PSM

		Before PSM				After PSM		
		ICG-LR n = 47	C-LR n = 100	P value		ICG-LR n = 40	C-LR n = 40	P value
Type of liver resection, n	Non-anatomical	18 (38)	44 (44)	0.063		16 (40)	19 (47)	0.499
	Anatomical	29 (62)	56 (56)			24 (60)	21 (53)	
	Segmentectomy	12	15			9	8	
	Sectionectomy	10	25			8	8	
	Major resection	7	16			7	5	
Surgical procedure, n	Open / laparoscopic	9 (19) / 38 (81)	33 (33) / 67 (67)	0.228		8 (20) / 32 (80)	12 (30) / 28 (70)	0.300
Surgical outcomes								
Operation time (min) §		405 (280–504)	398 (292–519)	0.998		401 (276–500)	343 (250–525)	0.351
Blood loss (ml) §		120 (0–260)	100 (10–300)	0.998		110 (3–258)	100 (10–278)	0.828
Blood transfusion, n		3 (6)	7 (7)	0.979		3 (7)	1 (2)	0.305
Weight of resected liver (g) §		140 (65–224)	150 (51–371)	0.721		146 (77–251)	135 (53–258)	0.433
Surgical margin, n	Positive cases	3 (6)	9 (9)	0.757		3 (7)	6 (15)	0.284
Postoperative blood examinations								
AST (IU/L) §		525 (292–693)	426 (224–718)	0.493		525 (272–664)	399 (200–785)	0.583
ALT (IU/L) §		429 (226–631)	382 (212–705)	0.239		433 (229–582)	401 (183–667)	0.683
Serum albumin (g/dL) §		2.6 (2.4–2.9)	2.6 (2.3–2.8)	0.333		2.7 (2.4–2.9)	2.6 (2.2–2.9)	0.277
Serum total bilirubin (mg/dL) §		1.4 (0.9–2.0)	1.5 (1.1–1.9)	0.753		1.4 (0.9–2.0)	1.4 (1.0–2.2)	0.579
Prothrombin time (%) §		72 (60–80)	63 (52–78)	0.184		73 (57–79)	63 (52–80)	0.439
Platelet count (10^3^/mm^3^) §		11.5 (9.3–16.2)	10.9 (8.2–16.0)	0.402		11.4 (9.3–16.4)	10.7 (8.1–14.3)	0.538
Postoperative outcomes								
Overall complication, n		17 (36)	33 (33)	0.539		14 (35)	14 (35)	1.000
Clavien-Dindo grade ≥ IIIa		4 (9)	15 (15)	0.007		3 (7)	5 (12)	0.456
Ascites		0	1			0	1	
Biliary leakage		1	11			0	3	
Intra-abdominal abscess		1	1			1	0	
Respiratory failure		1	0			1	0	
Pleural effusion		0	1			0	1	
Cerebral infraction		0	1			0	0	
Sick sinus syndrome		1	0			1	0	
Hospital stay (days) §		11 (9–14)	12 (9–17)	0.331		11 (10–14)	12 (9–15)	0.710

Values in parentheses are percentages unless indicated otherwise; §values are median (interquartile range)

Major resection was defined as the resection of three or more Couinaud segments

ALT, alanine transaminase; AST, aspartate aminotransferase; C-LR, conventional liver resection; ICG-LR, liver resection with ICG-fluorescence imaging; PSM, propensity score matching

Tables 5 and 6 show the comparisons of the baseline characteristics and surgical factors between the ICG-LR and C-LR groups of patients who underwent anatomical liver resection, respectively. In the entire cohort, the ICG-LR group comprised significantly more patients with underlying liver disease than did the C-LR group (Tables 5 and 55% vs. 27%, *P* = 0.010). The ICG-LR group had significantly higher serum total bilirubin levels (0.7 vs. 0.6 mg/dL; *P* = 0.016) and prothrombin time (103% vs. 97%; *P* = 0.047) than did the C-LR group. Regarding surgical factors, the ICG-LR group had a significantly lower Clavien–Dindo grade ≥ IIIa complication rate (Tables 6 and 7% vs. 25%; *P* = 0.043) and a shorter hospital stay (12 vs. 15 days; *P* = 0.012) than did the C-LR group. In the matched cohort, which included 21 patients in each group, there were no significant differences in patient background characteristics between the groups (Table 5). Regarding surgical factors, the ICG-LR group had a lower Clavien–Dindo grade ≥ IIIa complication rate (Table 6 and 0% vs. 24%; *P* = 0.017) and shorter hospital stays (12 vs. 14 days; *P* = 0.041) than the C-LR group did.

**Table 5:** Comparison of patient backgrounds between the ICG-LR and C-LR groups in patients who underwent anatomical liver resection before and after PSM

		Before PSM				After PSM		
		Anatomical ICG-LR n = 29	Anatomical C-LR n = 56	P value		Anatomical ICG-LR n = 21	Anatomical C-LR n = 21	P value
General background								
Age (y) §		72 (68–80)	72 (66–77)	0.871		72 (61–80)	72 (66–76)	0.787
Sex, n	Male / Female	21 (72) / 8 (28)	45 (80) / 11 (20)	0.410		15 (71) / 6 (29)	12 (57) / 9 (43)	0.333
BMI (kg/m^2^) §		23.6 (20.7–24.8)	23.4 (20.6–25.9)	0.767		23.1 (20.7–24.6)	20.7 (18.8–24.9)	0.320
Performance status, n	0	27 (93)	50 (89)	0.586		20 (95)	19 (90)	0.220
	1	2 (7)	4 (7)			1 (5)	0 (0)	
	2	0 (0)	2 (4)			0 (0)	2 (10)	
Underlying liver disease, n	HB, HC	16 (55)	15 (27)	0.010		9 (43)	8 (38)	0.753
	non-B non-C	13 (45)	41 (73)			12 (57)	13 (62)	
Child-Pugh classification, n	A / B	29 (100) / 0 (0)	54 (96) / 2 (4)	0.303		21 (100) / 0 (0)	20 (95) / 1 (5)	0.312
Blood examination								
AST (IU/L) §		25 (22–44)	30 (22–46)	0.565		34 (22–49)	26 (21–41)	0.597
ALT (IU/L) §		26 (16–54)	27 (16–40)	0.867		26 (18–56)	23 (16–36)	0.358
Serum albumin (g/dL) §		4.1 (3.9–4.4)	4.0 (3.7–4.5)	0.332		4.1 (3.7–4.4)	4.2 (3.7–4.6)	0.588
Serum total bilirubin (mg/dL) §		0.7 (0.6–1.1)	0.6 (0.4–0.9)	0.016		0.7 (0.6–1.0)	0.6 (0.5–1.0)	0.335
Prothrombin time (%) §		103 (95–109)	97 (85–106)	0.047		103 (96–106)	105 (93–109)	0.538
Platelet count (10^3^/mm^3^) §		20.8 (16.6–24.0)	20.4 (16.1–27.9)	0.982		21.9 (18.6–25.2)	20.8 (16.6–28.0)	0.841
ICGR15 (%) §		9.8 (7.1–13.8)	9.2 (7.1–13.0)	0.704		9.1 (7.6–12.8)	8.3 (6.9–11.6)	0.421
Tumor factor								
Diagnosis, n	HCC	22 (76)	43 (77)	0.219		14 (67)	15 (71)	0.169
	Metastatic tumor	3 (10)	11 (20)			3 (14)	6 (29)	
	ICC	3 (10)	2 (3)			3 (14)	0 (0)	
	Others	1 (4)	0 (0)			1 (5)	0 (0)	
Tumor size (cm) §		4.8 (3.4–6.5)	5.1 (3.5–9)	0.467		5.0 (3.4–6.8)	4.0 (3.1–6.5)	0.320
Tumor number, n	1 / ≥ 2	22 (76) / 7 (24)	31 (55) / 25 (45)	0.060		15 (71) / 6 (29)	11 (52) / 10 (48)	0.202
Macrovascular invasion, n		6 (21)	15 (27)	0.533		6 (29)	6 (29)	1.000

Values in parentheses are percentages unless indicated otherwise; §values are median (interquartile range)

ALT, alanine transaminase; AST, aspartate aminotransferase; BMI, body mass index; C-LR, conventional liver resection; HB, hepatitis B; HC, hepatitis C; HCC, hepatocellular carcinoma; ICC, intrahepatic cholangiocarcinoma; ICGR15, indocyanine green retention rate at 15 min; ICG-LR, liver resection using ICG-fluorescence imaging; NA, not applicable; non-B non-C, nonhepatitis B and nonhepatitis C; PSM, propensity score matching

**Table 6:** Comparison of surgical factors between the ICG-LR and C-LR groups in patients who underwent anatomical liver resection before and after PSM

		Before PSM				After PSM		
		Anatomical ICG-LR n = 29	Anatomical C-LR n = 56	P value		Anatomical ICG-LR n = 21	Anatomical C-LR n = 21	P value
Type of liver resection, n	Segmentectomy	12 (41)	15 (27)	0.393		9 (43)	6 (29)	0.291
	Sectionectomy	10 (34)	25 (45)			6 (29)	11 (52)	
	Major resection	7 (24)	16 (29)			6 (29)	4 (19)	
Surgical procedure, n	Open / laparoscopic	9 (31) / 20 (69)	29 (52) / 27 (48)	0.065		6 (29) / 15 (71)	8 (38) / 13 (62)	0.512
Surgical outcomes								
Operation time (min) §		461 (374–531)	443 (342–544)	0.978		429 (321–515)	417 (339–551)	0.950
Blood loss (ml) §		200 (65–353)	200 (100–473)	0.587		150 (35–353)	145 (55–330)	0.840
Blood transfusion, n		3 (10)	6 (11)	0.958		2 (10)	2 (10)	1.000
Weight of resected liver (g) §		216 (126–476)	255 (163–633)	0.147		224 (145–490)	251 (153–469)	0.782
Surgical margin, n	Positive cases	2 (7)	4 (7)	0.967		2 (10)	2 (10)	1.000
Postoperative blood examinations								
AST (IU/L) §		563 (343–846)	501 (353–841)	0.721		553 (343–690)	534 (317–827)	0.763
ALT (IU/L) §		511 (251–764)	425 (246–760)	0.453		508 (251–665)	433 (279–745)	0.831
Serum albumin (g/dL) §		2.6 (2.4–2.9)	2.5 (2.2–2.7)	0.064		2.6 (2.4–2.9)	2.4 (2.3–2.7)	0.080
Serum total bilirubin (mg/dL) §		1.6 (1.3–2.3)	1.6 (1.2–2.0)	0.867		1.5 (1.2–2.0)	1.6 (1.2–2.0)	0.801
Prothrombin time (%) §		65 (52–76)	58 (45–68)	0.151		70 (53–76)	60 (53–77)	0.589
Platelet count (10^3^/mm^3^) §		10.9 (9.0–13.6)	10.9 (7.9–16.2)	0.813		11.7 (10.3–17.1)	11.4 (8.2–20.5)	0.821
Postoperative outcomes								
Overall complication, n		15 (52)	25 (45)	0.756		9 (43)	12 (57)	0.354
Clavien-Dindo grade ≥ IIIa		2 (7)	14 (25)	0.043		0 (0)	5 (24)	0.017
Ascites		0	1			0	1	
Biliary leakage		1	11			0	3	
Intra-abdominal abscess		0	1			0	0	
Pleural effusion		0	1			0	1	
Sick sinus syndrome		1	0			0	0	
Hospital stay (days) §		12 (10–14)	15 (10–25)	0.012		12 (11–13)	14 (10–33)	0.041

Values in parentheses are percentages unless indicated otherwise; §values are median (interquartile range)

Major resection was defined as the resection of three or more Couinaud segments

ALT, alanine transaminase; AST, aspartate aminotransferase; C-LR, conventional liver resection; ICG-LR, liver resection using ICG-fluorescence imaging; PSM, propensity score matching

### Comparison of the short-term prognosis between the ICG-LR and C-LR groups in patients with HCC

**Fig. 1:**
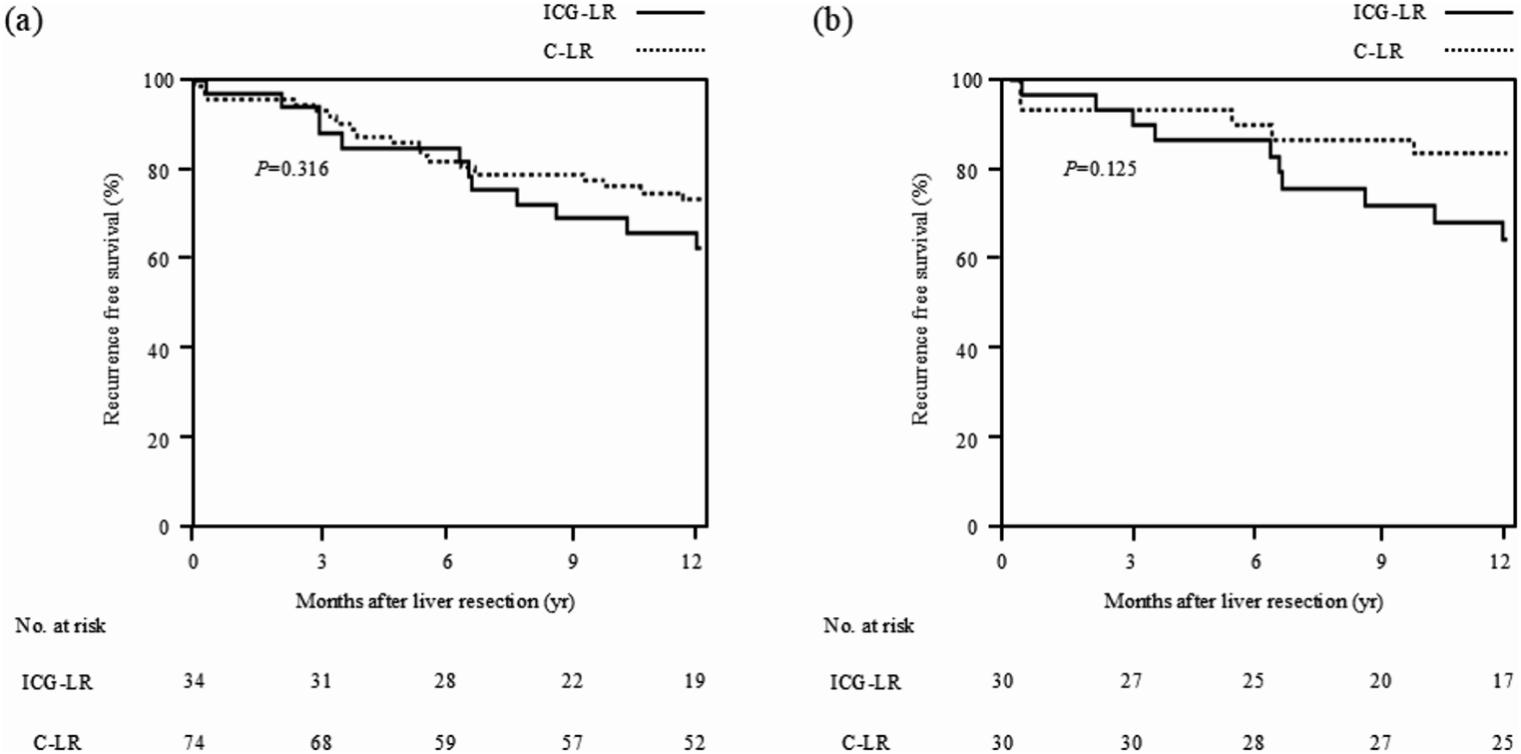
Comparison of 1-year RFS between the ICG-LR and C-LR groups in patients with HCC. (**a**) In the entire cohort, the 1-year RFS rates after liver resection were 63% and 73% in the ICG-LR and C-LR groups in patients with HCC, respectively; there were no significant difference between groups (*P* = 0.316). (**b**) In the matched cohort, the 1-year RFS rates after liver resection were 64% and 83% in the ICG-LR and C-LR groups in patients with HCC, respectively; there were no significant differences between groups (*P* = 0.125). C-LR, conventional liver resection; HCC, hepatocellular carcinoma; ICG, indocyanine green; ICG-LR, liver resection using ICG-fluorescence imaging; RFS, recurrence free survival

The results of the comparison of 1-year RFS rate between the ICG-LR and C-LR groups in patients with HCC are shown in Fig. 1. There were 34 and 74 patients with HCC in the ICG-LR and C-LR groups, respectively. The 1-year RFS rates after liver resection were 63% and 73% in the ICG-LR and C-LR groups in patients with HCC, respectively, demonstrating no significant between-group difference (*P* = 0.316). After PSM, 30 patients were included in each of these groups. The 1-year RFS rates after liver resection were 64% and 83% in the ICG-LR and C-LR groups in patients with HCC, respectively, also demonstrating no significant difference (*P* = 0.125).

## Discussion

The efficacy and safety of ICG-LR was expected to be further clarified in this study. Our findings revealed that ICG-LR was safely performed with a 60% identification rate of hepatic boundaries during liver resection. When surgical outcomes were compared between the ICG-LR and C-LR groups by focusing on anatomical liver resection, the ICG-LR group had a significantly lower rate of severe liver-associated complications than the C-LR group did. These results indicate that ICG-fluorescence imaging could be used to recognize hepatic boundaries during liver transection and may enhance the safety of anatomical liver resection.

The use of ICG is definitely a breakthrough in the development of liver resection technology. Makuuchi et al. were first to conceptualize an identification technique for hepatic boundaries using ICG injection through the portal vein during liver resection [17]. In parallel with advancements in imaging technology for liver resection, ICG-fluorescence imaging has been aggressively used during this procedure [4, 9, 18]. Among several applications of ICG-LR, the potential for identifying intrahepatic boundaries during resection, which cannot be performed using conventional methods, is one of its practical advantages [5–7, 9, 11, 12]. The three-dimensional intrahepatic resection plane, conventionally determined based on intrahepatic landmarks and empirical images from liver surgeons, is now identified using ICG-fluorescence imaging, which highlights the demarcation between fluorescing and non-fluorescing areas. Here, we first validated the identification rate of hepatic boundaries, from the perspective of both the hepatic surface and intrahepatic boundary, by ICG-fluorescence imaging. Consequently, based on the definition used in this study, the hepatic boundaries were recognized in 60% of patients who underwent ICG-LR (Table 2). A recent systematic review of 72 articles showed that the successful segmentation rate was 88.0% (range, 53–100%) [10]. The low identification rate of hepatic boundaries found in this study is probably due to the relatively complicated criteria used for the successful identification of hepatic boundaries and the inclusion of cases in which non-anatomical liver resection was performed. When focusing on cases in which anatomical liver resection was performed, the successful identification rate was 72%. Accordingly, we considered ICG-LR to be a practical method for the recognition of intrahepatic boundaries, especially in cases of anatomical liver resection.

Since ICG-fluorescence imaging was introduced in the field of liver surgery, many researchers have investigated the efficacy of the system by focusing on its technical aspects, including the intraoperative detection of liver tumors and hepatic boundaries [4–9]. Recently, its advantages over conventional methods in terms of surgical outcomes have been gradually recognized [19–23]. Among them, it has often been reported that the number of cases with negative surgical margins was significantly higher in the ICG-LR group than in the C-LR group [19, 20, 22]. Additionally, Liu et al. reported that the ICG-LR group had significantly better RFS than the C-LR group did [22]. Thus, it is possible that ICG-LR has some advantages over C-LR in terms of surgical outcomes. However, most studies have retrospectively evaluated the surgical results. Furthermore, the definition of hepatic boundaries has not been consistent among reports. Therefore, it is difficult to claim that the advantages of ICG-LR over C-LR in terms of the surgical outcomes of liver surgery have been sufficiently investigated. In this study, we compared the surgical outcomes between ICG-LR and C-LR using prospective data and a concrete definition of the successful identification of hepatic boundaries. Overall, we did not observe a significant superiority of any surgical outcome in the ICG-LR group compared with the C-LR group (Tables 3 and 4). However, when we focused on anatomical liver resection, which was associated with the successful identification of hepatic boundaries using the negative staining technique of ICG-fluorescence imaging (data not shown), Clavien–Dindo grade ≥ IIIa complications occurred significantly less frequently in the ICG-LR group than in the C-LR group (Table 6). The observed Clavien–Dindo grade ≥ IIIa complications included ascites in one patient and bile leak in three patients, all of which were liver-associated. This suggests that ICG-LR facilitated the accurate execution of liver resection with less liver damage. Additionally, navigated liver resection would be useful for confirming the direction of parenchymal resection, possibly reducing stress levels in liver surgeons. It may also help compensate for the lack of experience among liver surgeons by indicating the proper direction of parenchymal resection in a three-dimensional space. To confirm the advantages of ICG-LR, further investigations using a larger cohort are warranted.

In this study, we also assessed the usefulness of ICG-fluorescence imaging for detecting liver tumors. The detection rate of liver tumors is reportedly 87.4% (range, 43 − 100%) [10]. However, in this study, it was relatively lower at 60%. This discrepancy could be explained by the assessment criteria we used for the successful identification of liver tumors. In this regard, the tumor location was not considered, although the tissue penetration of the fluorescence emitted by ICG was only approximately 5–10 mm [24]. When we focused on the liver tumors located around the liver surface, the detection rate reached 84%. Accordingly, we considered that our study confirmed the usefulness of ICG-fluorescence imaging for detecting superficial liver tumors.

We investigated the safety of ICG-LR from both surgical and oncological aspects. No adverse events related to ICG injections were observed. The incidence rates of severe complications were lower in the ICG-LR group than in the C-LR group (Table 6). As for oncological aspects, the number of positive surgical margins did not significantly differ between groups. Furthermore, the 1-year RFS rates were similar between the ICG-LR and C-LR groups in patients with HCC (Fig. 1). These results indicate that we could perform ICG-LR with better surgical and similar oncological safety as compared with C-LR.

This study had several limitations, including its single-center design and small sample size. The number of patients did not reach the planned sample size owing to the limited registration duration. Therefore, further investigations using a larger cohort are warranted to confirm the results of this study. As for the method used, ICG-fluorescence imaging was performed exclusively using the negative staining technique. Although this technique is more suitable for anatomical liver resection than for partial liver resection, we included patients who underwent partial liver resection because the procedure was performed based on the concept of cone unit resection [16]. The successful identification rate of hepatic boundaries and results of the comparison of surgical outcomes between the ICG-LR and C-LR groups may have been affected by the inclusion of cases in which partial liver resection was performed. Additionally, the learning curve in terms of the ICG-fluorescence imaging technique could have affected the successful identification of hepatic boundaries owing to the short duration of this study, possibly leading to a lower rate of successful identification of hepatic boundaries. Finally, although our study was prospective, we retrospectively reviewed the data of patients who underwent C-LR and compared surgical outcomes between the ICG-LR and C-LR groups.

## Conclusion

ICG-fluorescence imaging could be used to recognize hepatic boundaries during liver transection. Additionally, ICG-LR may be useful in preventing severe liver-associated complications in patients undergoing anatomical liver resection. Further investigation to clarify the advantages of ICG-LR in terms of the postoperative surgical outcomes of liver resection is warranted.

## Electronic supplementary material

Below is the link to the electronic supplementary material.

Supplementary Material 1

## Data Availability

The Registration Data Set is available at https://jrct.niph.go.jp/.
