# Supplementary figures and images for: Efficacy and safety of indocyanine green-fluorescence imaging guided liver resection: a single-arm prospective cohort study
Source: Langenbecks Arch Surg. 2025 Jan 11;410(1):34. doi: 10.1007/s00423-024-03602-7 (PMC11723889; doi:10.1007/s00423-024-03602-7)

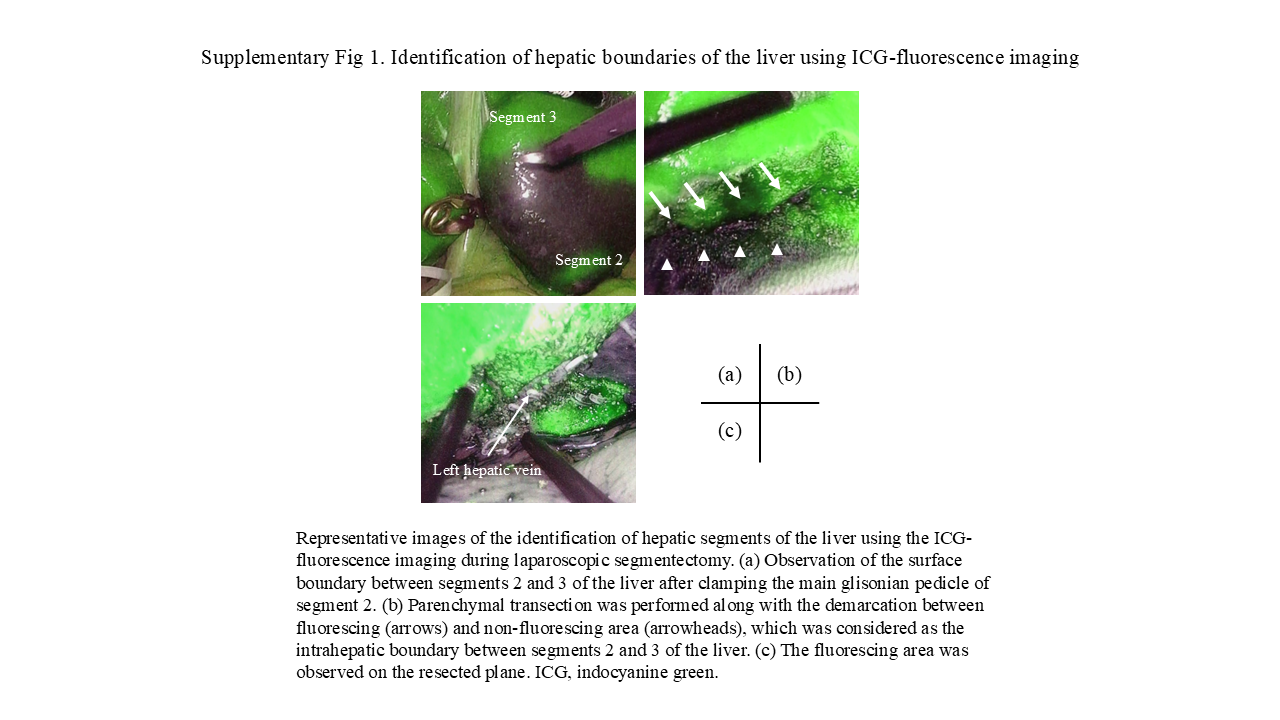

Supplement: Supplementary file 1 — Supplementary Material 1 [file 423_2024_3602_MOESM1_ESM.tif]
